# Internal comparison between deuterium oxide (D_2_O) and L*-*[*ring*-^13^C_6_] phenylalanine for acute measurement of muscle protein synthesis in humans

**DOI:** 10.14814/phy2.12433

**Published:** 2015-07-06

**Authors:** Daniel J Wilkinson, Jessica Cegielski, Bethan E Phillips, Catherine Boereboom, Jonathan N Lund, Philip J Atherton, Kenneth Smith

**Affiliations:** MRC-ARUK Centre of Excellence for Musculoskeletal Ageing Research, School of Medicine, University of NottinghamDerby, UK

**Keywords:** Deuterium oxide, metabolism, muscle protein synthesis, stable isotope tracers

## Abstract

Stable isotope tracer methodologies are becoming increasingly widespread in metabolic research; yet a number of factors restrict their implementation, such as, i.v infusions, multiple cannulae, tissue samples, and significant cost. We recently validated the sensitivity of the orally administered stable isotope tracer deuterium oxide (D_2_O) for quantifying day-to-day changes in muscle protein synthesis (MPS). This method is less invasive, restrictive, and more cost-effective than traditional amino acid (AA) tracer techniques. In the present study, we hypothesized the sensitivity of our analytical techniques (GC-Pyrolysis-IRMS) would permit D_2_O-derived measurements of MPS over much shorter periods (i.e., hours) usually only possible using AA-tracer techniques. We recruited nine males (24 ± 3 year, BMI: 25 ± 3 kg·m^−^²) into an internally controlled comparison of D_2_O versus ^13^C AA-tracers. The day before the acute study subjects consumed 400 mL D_2_O, and on the study day, received a primed (0.3 mg·kg^−1^) continuous (0.6 mg·kg·h^−1^) i.v infusion of L-[*ring*-^13^C_6_]-phenylalanine to quantify MPS under both: (1) basal [postabsorptive] and; (2) stimulated [postprandial] that is, consumption of 20 g EAA, conditions. Measures of MPS yielded indistinguishable technique differences with respect to EAA, ^13^C: 0.065 ± 0.004 to 0.089 ± 0.006%·h^−1^ (*P* < 0.05) and D_2_O: 0.050 ± 0.007 to 0.088 ± 0.008%·h^−1^ (*P* < 0.05) with qualitatively similar increases. Our findings reveal that acute measurement of MPS, usually only possible using AA-tracers, are feasible over shorter periods with orally administered D_2_O when used in tandem with GC-Pyrolysis-IRMS. We conclude that this D_2_O approach provides a less invasive, cost-effective, and flexible means by which to quantify MPS acutely over several hours.

## Introduction

Stable isotope tracers have been at the forefront of metabolic research since the successful isolation of the stable isotope deuterium by Harold Urey in 1931 (Urey et al. 1932). This combined with the rapid and dedicated development of these new techniques over subsequent years by Urey, Schoenheimer, and Rittenberg, has provided us with an exquisite array of tools for understanding metabolic systems and substrate turnover (Schoenheimer and Rittenberg 1935, 1938; Rittenberg and Schoenheimer 1937; Rennie et al. 1982; Zhang et al. 1996, 2012; Wolfe and Chinkes 2005). Such isotopic methodologies have been key to our understanding of the regulation of skeletal muscle mass in particular (Atherton and Smith 2012). With the diversity of muscle functions ranging from; the control of locomotion and physical function to acting as a fuel reservoir for other organs in times of stress/illness, maintenance of muscle mass is essential for ensuring metabolic health, in addition to maintaining independence and quality-of-life into older age.

Over recent decades, the study of human muscle protein turnover has been aided through the use of stable isotopically labeled amino acids (AA) with either heavy carbon (^13^C), hydrogen (^2^H) or nitrogen (^15^N) motifs. The incorporation of these labeled AA into muscle protein permits the calculation of a fractional synthetic rate (FSR) of muscle protein synthesis (MPS). Whilst monitoring of the rate of disappearance of the tracer from the arterial pool, and appearance of the tracer in the venous pool across a specified tissue provides rates of synthesis and breakdown respectively. However, these approaches have limitations. Application of these tracers often requires complex experimental set-up involving i.v infusions, which necessitate venous (and arterial) cannulation, alongside multiple biopsies and are generally restricted to periods of up to 12 h. Such limitations make it difficult to apply these techniques to certain populations (frail elderly, critical care, CKD, young children and adolescents) and also restrict the acquisition of longer term, potentially more habitually relevant metabolic readouts.

We have recently demonstrated that by providing a single oral bolus of the tracer deuterium oxide (D_2_O), we were able to accurately measure MPS over a period of as little as 2 days both at rest and in response to exercise (Wilkinson et al. 2014). This method is far less invasive, requiring only a single tissue [muscle] biopsy, and has been used to measure MPS over periods between 1 or 2 days (Gasier et al. 2012; Wilkinson et al. 2014)) to several weeks (Robinson et al. 2011). Due to the sensitivity of the methods we pioneered, that is, GC-pyrolysis-IRMS, we predicted we would be able to detect MPS differences over even shorter periods of several hours (∼3–6 h), a time period generally only conducive to the use of traditional AA-tracers. If true, this would provide an alternative for the measurement of MPS without the need for invasive routes of tracer administration or multiple-blood and tissue sampling in a more cost-effective and less invasive manner. As such, the aim of this study was to internally compare our D_2_O tracer technique against traditional AA-tracer techniques using L-[*ring*-^13^C_6_] phenylalanine; we quantified MPS using both tracers under basal (postabsorptive) and stimulated (postprandial) conditions. A comparison between phenylalanine tracers and D_2_O for the measurement of acute MPS has been performed previously (Gasier et al. 2009), however, rats turnover approximately 10-fold faster than humans, additionally there were distinct differences between measurement periods employed (4 h and 24 h for D_2_O and 12 mins for Phenylalanine) and mode of tracer administration (phenylalanine was administered as a flooding dose rather than primed constant infusion). In the present study, we performed an internal comparison in humans between L-[*ring*-^13^C_6_] phenylalanine and D_2_O within the same individuals using the same design and measurement periods, in order to determine whether D_2_O could provide a robust alternative, and simpler, less burdensome approach for use in laboratories and populations where repeat invasive sampling and i.v tracer administration may not be possible.

## Materials and Methods

### Subject characteristics and ethics

Nine healthy young males (24 ± 3 year, BMI: 25 ± 3 kg·m^−^²) were recruited. All subjects were screened by means of a medical questionnaire, physical examination, and resting ECG, with exclusions for metabolic, respiratory, and cardiovascular disorders or other symptoms of ill-health. Subjects had normal blood chemistry, were normotensive (BP < 140/90) and were not prescribed any medications; all subjects performed activities of daily living and recreation. All subjects gave their written, informed consent to participate after all procedures and risks were explained. This study was approved by The University of Nottingham Ethics Committee and complied with the Declaration of Helsinki.

### Study procedures

**Figure 1:**
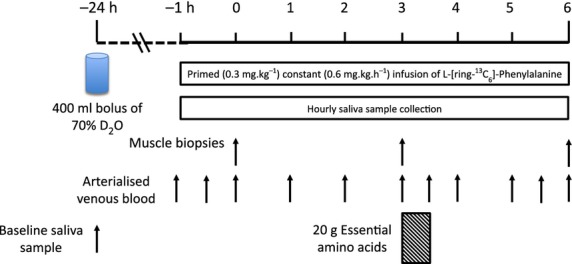
Schematic of D_2_O L-[*ring*-^13^C_6_]-Phenylalanine dual tracer comparison study protocol.

Subjects were asked to refrain from heavy exercise for 72 h before the start of the study. About 24 h prior to the start of the study, following provision of a basal saliva sample, each subject was provided with 400 mL D_2_O (70 Atom%, Sigma Aldrich, Poole, UK), which was consumed in four doses of 100 mL at 2 h intervals from 2 pm that afternoon (to avoid potential side effects associated with consumption of large doses of D_2_O, e.g., dizziness, nausea). The subjects were advised to consume their normal diet that evening and following an overnight fast (>10 h), subjects arrived to the laboratory at ∼0830 h the following day and had an 18 g cannula inserted into the antecubital vein of one arm for tracer infusion and a retrograde 14 g cannula inserted to sample arterialized blood from the dorsal capillary bed of the opposite hand (using the ‘hot hand’ method; (Greenhaff et al. 2008)). A primed, continuous infusion (0.3 mg·kg^−1^ prime, 0.6 mg·kg·h^−1^ continuous infusion) of L-[*ring*-^13^C_6_]-phenylalanine (99 Atoms %, Cambridge Isotopes Limited, Cambridge, MA) was started (at *t* = −1 h) and maintained until the end of the study (6 h). 1 h after the beginning of the infusion the first biopsy was collected (0 h) with a second biopsy collected 3 h later (3 h). This allowed us to gather “basal”, postabsorptive measurements (0–3 h), before subjects consumed 20 g of essential amino acids (EAA; see Table1 for composition, with 6% of the phenylalanine provided as L-[*ring*-^13^C_6_]-phenylalanine to prevent dilution of the tracer) dissolved in 250 mL of water for measures of postprandial MPS from 3–6 h, with the third and final biopsy collected at 6 h. Muscle biopsies (∼200 mg) were taken from *m. vastus lateralis* under sterile conditions using local anesthetic (1% lidocaine). A schematic of the protocol is shown in Fig.1. In addition to blood and muscle, subjects provided saliva throughout (inclusive of a sample prior to oral administration of D_2_O) for the measurement of body water enrichment.

**Table 1:** Composition of essential amino acid drink.

Essential amino acid	Mass added (g)	Percentage mass added (%)
Histidine	1.61	8.09
Isoleucine	2.31	11.60
Leucine	4.79	24.06
Lysine	4.09	20.54
Methionine	1.27	6.38
Phenylalanine	1.21	6.08
Threonine	1.51	7.58
Tryptophan	0.64	3.21
Valine	2.48	12.46

### Body water enrichment

Water was obtained from saliva samples prior to analysis. 100 *μ*L aliquots of saliva were placed in the cap of inverted auto-sampler vials; these were then placed on a heating block at 90°C for 4 h. Water distillate was collected by rapidly cooling the vials on ice for 10 min, and was transferred to fresh vials for direct liquid injection into a high temperature conversion elemental analyzer (TC/EA; Thermo Scientific, Hemel Hempstead, UK) connected to an Isotope Ratio Mass Spectrometer (IRMS, Delta V Advantage; Thermo Scientific) as previously described Wilkinson et al. (2014).

### Isolation and derivatization of myofibrillar protein fractions

For isolation of myofibrillar fractions ∼30–50 mg of muscle was used. The muscle was homogenized in ice-cold homogenization buffer (50 m·molL^−1^ Tris-HCl (pH 7.4), 50 m·molL^−1^ NaF, 10 m·molL^−1^ *β*-Glycerophosphate disodium salt, 1 m·molL^−1^ EDTA, 1 m·molL^−1^ EGTA, 1 m·molL^−1^ activated Na_3_VO_4_ (all Sigma-Aldrich)) and a complete protease inhibitor cocktail tablet (Roche, West Sussex, UK) at 10 *μ*L·*μ*g^−1^ of tissue. Homogenates were rotated for 10 min and the supernatant collected by centrifugation at 10,000 × *g* for 5 min at 4°C. The myofibrillar pellets were solubilized in 0.3 molL^−1^ NaOH, separated from insoluble collagen by centrifugation and the myofibrillar protein precipitated with 1 molL^−1^ perchloric acid (PCA). Protein-bound AA were released using acid hydrolysis by incubating in 0.1 molL^−1^ HCl in Dowex H^+^ resin slurry overnight at 110°C before being eluted from the resin with 2 molL^−1^ NH_4_OH and evaporated to dryness. The AA were then derivatized as their n-methoxycarbonyl methyl esters (MCME) according to the protocol of Husek (Husek and Liebich 1994) with slight modifications. The dried samples were resuspended in 60 *μ*L of distilled water and 32 *μ*L of methanol, following a brief vortex, 10 *μ*L of pyridine and 8 *μ*L of methylchloroformate were added. Samples were vortexed for 30 sec and left to react at room temperature for 5 min. Newly formed MCME AA were then extracted into 100 *μ*L of chloroform; any remaining water was removed from the sample with addition of a molecular sieve. Incorporation of deuterium into protein-bound alanine was determined by gas chromatography-pyrolysis-isotope ratio mass spectrometry, and the incorporation of L-[*ring-*^13^C_6_] phenylalanine into myofibrillar protein by gas chromatography-combustion-isotope ratio mass spectrometry. Both analyses were performed using the same system (Delta V Advantage; Thermo Scientific) utilizing a GC isolink and four port switching-valve to permit switching between pyrolysis and combustion ovens.

### Plasma/intracellular phenylalanine labeling and plasma BCAA concentrations

For plasma AA concentration and enrichment measurements, proteins were precipitated with 100% ethanol following addition of 10 *μ*L of norleucine internal standard (250 *μ*g·mL^−1^) and the supernatant evaporated to dryness, reconstituted in 0.5 molL^−1^ HCl and the lipid fraction removed using ethyl acetate extraction. The remaining aqueous phase was then evaporated to dryness. For intracellular enrichment, 200 *μ*L of the sarcoplasmic fraction was deproteinized with 1 molL^−1^ PCA, the supernatant was neutralized and purified on Dowex H^+^ resin before being evaporated to dryness. Plasma and intracellular phenylalanine, and plasma leucine, isoleucine and valine were then converted to their tBDMS derivative and enrichment (APE) determined using gas chromatography-mass spectrometry (Trace 1300 - ISQ, GC-MS and Trace 1310 – TSQ, GC-MS/MS; Thermo Scientific) in single ion monitoring mode. Plasma BCAA concentrations were determined with reference to a standard curve.

### Calculation of fractional synthetic rate (FSR)

The FSR of myofibrillar proteins (MPS) using the D_2_O tracer was determined, using the precursor-product approach, from the incorporation of deuterium labeled alanine into protein, using the enrichment of body water (corrected for the mean number of deuterium moieties incorporated per alanine, 3.7, and the total number of hydrogen within the alanine derivative, 11) as the surrogate precursor labeling between subsequent biopsies. In brief, the standard equation is: 

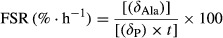


where, *δ*_Ala_ = deuterium enrichment (in delta excess) of protein-bound alanine between subsequent biopsies, *δ*_P_ = precursor enrichment (in delta excess) and *t*, time between biopsies.

Fractional synthetic rate (FSR) of myofibrillar proteins using the L-[*ring-*^13^C_6_]-phenylalanine tracer was calculated using the same standard precursor-product equation as above: 

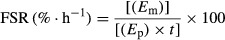


where *E*_m_ = the change in the protein bound L-[*ring*-^13^C_6_]-phenylalanine enrichment in atoms per excess (APE) between subsequent biopsies and *E*_p_ = the mean enrichment over the same time period (*t*) of the precursor for protein synthesis (calculated from the mean of the two intracellular L-[*ring-*^13^C_6_]-phenylalanine enrichment (APE) over the defined measurement period).

### Statistical analyses

Descriptive statistics were produced for all data sets to check for normal distribution (accepted if *P* > 0.05) using a Kolmogorov–Smirnov test. Differences within groups and between groups (feed × tracer) were detected by two-way ANOVA with a Sidak-Holm post hoc test using GraphPad Prism software (Version 5, La Jolla, San Diego CA). Limits of agreement between methods were performed using Bland–Altman plots, with correlations analyzed using Pearson’s Product Moment coefficient. The alpha level of significance was set at *P* < 0.05. All data are presented as mean ± SEM.

## Results

### Plasma AA concentrations

**Figure 2:**
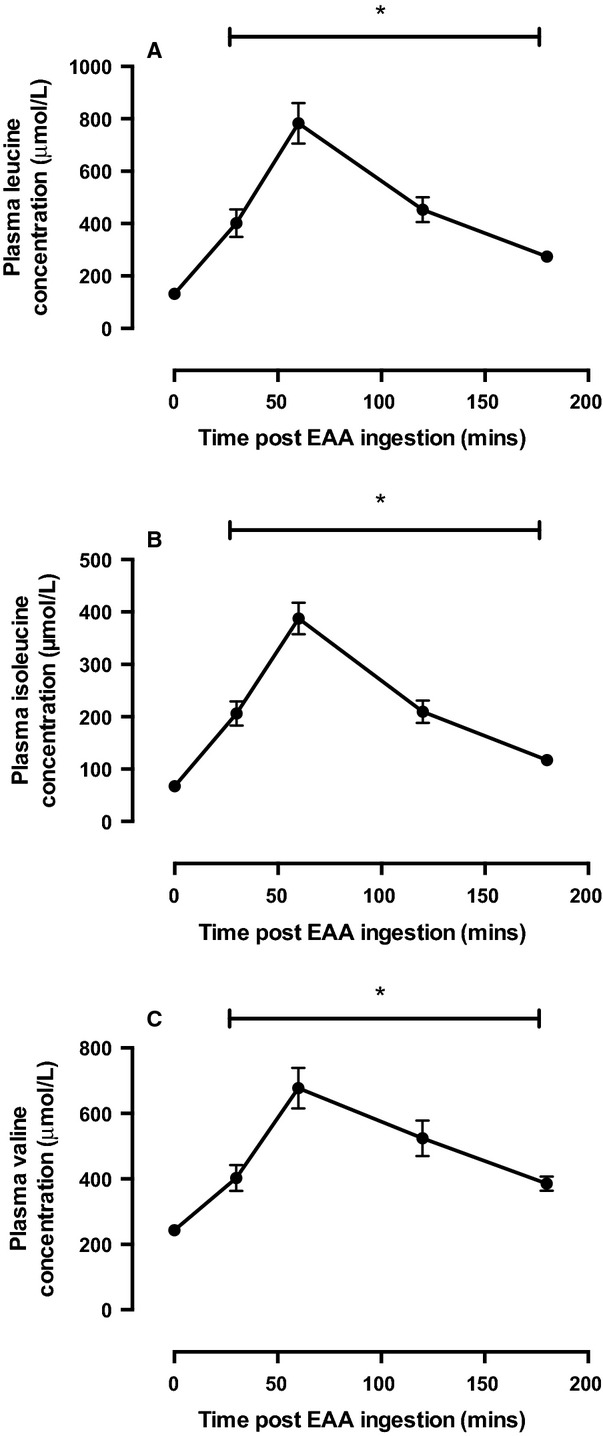
Plasma (A) Leucine (B) Isoleucine, and (C) Valine concentrations. *Significantly different from fasted *P* < 0.05.

As would be expected, we observed significant increases in plasma AA concentrations following EAA consumption as represented by the concentrations of leucine, valine, and isoleucine (*P* < 0.01; Fig.2). Concentrations of AA peaked ∼60 min following feeding and were significantly different from fasted at all postprandial time points during the study period.

### Body water, plasma, and intracellular phenylalanine enrichment

**Figure 3:**
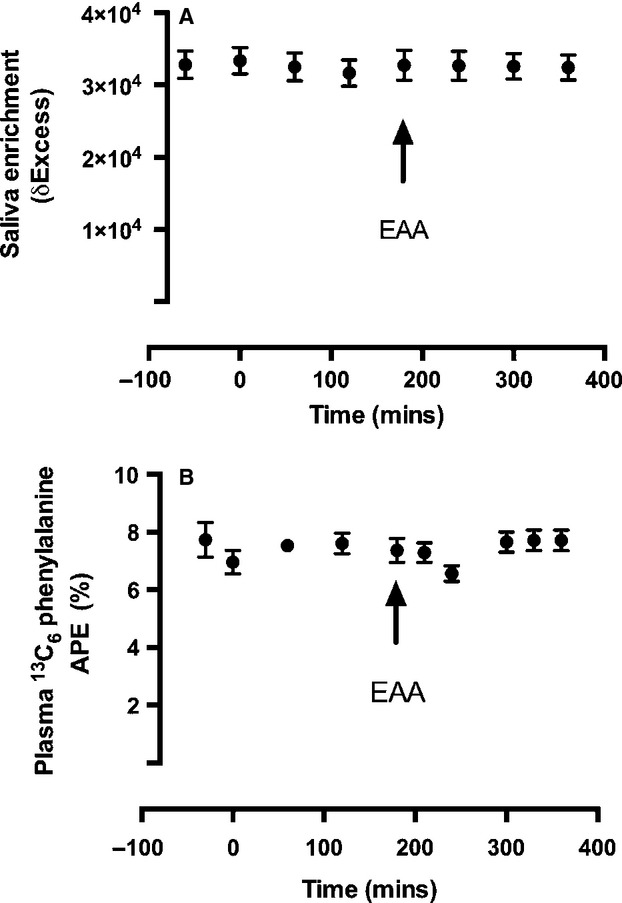
(A) Saliva body water enrichment in delta excess notation and (B) plasma phenylalanine enrichment in APE.

Body water enrichments were ∼33,000 *δ* and due to the slow turnover rate of body water this level of enrichment maintained a pseudo-steady state throughout the acute study period (see Fig.3). Plasma L-[*ring-*^13^C_6_] phenylalanine enrichment also maintained a steady state throughout the study period. Mean intracellular L-[*ring-*^13^C_6_] phenylalanine enrichment for the postabsorptive and postprandial periods were 4.1 ± 0.5 and 4.75 ± 0.2 APE respectively.

### Myofibrillar protein synthesis: comparison of D_2_O and L-[ring-^13^C_6_]-phenylalanine tracers

**Figure 4:**
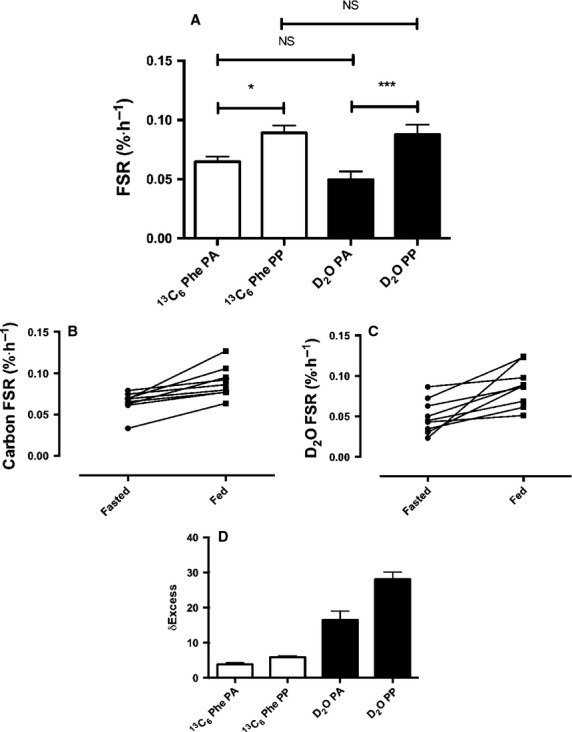
(A) Comparison of mean postabsorptive (PA) and postprandial (PP) myofibrillar FSR as measured using D_2_O (black bars) and L-[ring-^13^C_6_]-phenylalanine (clear bars). *P < 0.05; ***P < 0.001. (B) Individual carbon FSR values, and (C) Individual D_2_O FSR values. (D) Absolute *δ* excess values for carbon and deuterium tracers.

**Figure 5:**
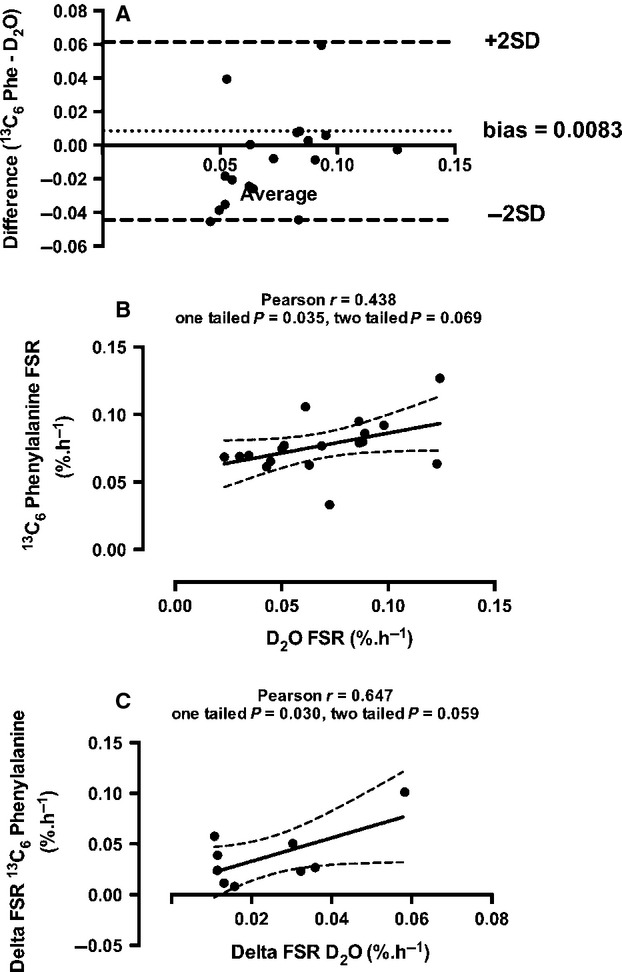
(A) Bland–Altman plot of comparison between the two tracer methods, (B) Correlation between FSR values produced using each tracer method, (C) Correlation between the change in FSR due to anabolic stimulus (EAA feed).

Postabsorptive rates of MPS were 0.050 ± 0.007 and 0.065 ± 0.004%·h^−1^, respectively, using the D_2_O and L-[*ring*-^13^C_6_]-phenylalanine tracers (Fig.4A). Following consumption of the EAA solution, rates of MPS increased to 0.088 ± 0.008 and 0.089 ± 0.006%·h^−1^ respectively, this was significantly higher than postabsorptive rates for each tracer (Fig.4A). Despite the qualitatively similar means-based data, if each subject is investigated individually and the two tracer techniques are directly compared quantitatively, the rates produced from the two techniques did not always yield the same differences (Fig.4B and C). A Bland–Altman plot of the agreement between the two methods can be observed in Fig.5A, with the calculated bias between the two methods 0.0083, suggesting that overall the two methods agree reasonably well, however, it can be observed from the plot that a small number of data points do not agree so well with one falling outside the 95% confidence intervals. Despite this, there was a significant correlation between FSR for the two methods; Pearson *r* = 0.438, one tailed *P* = 0.035, two tailed *P* = 0.069 (Fig.5B), as well as the delta FSR change from postabsorptive to postprandial (Fig.5C).

## Discussion

The requirement for complex clinical experimental set-up, alongside the need for invasive cannulation and biopsy techniques limits the application of acute AA-tracer techniques; especially within certain populations. Here, we show that it is possible to quantify MPS over several hours without the need for cannulation or the iv infusion of expensive AA-tracers. Using an oral dose of D_2_O, we demonstrate that with collection of saliva and muscle samples it is possible to accurately quantify MPS over as short a period as 3 h in the postabsorptive state, whilst also being able to detect significant increases in MPS in the postprandial state. Furthermore, this technique produced comparable mean rates to that obtained when using traditional L-[*ring*-^13^C_6_]-phenylalanine tracers in the same individuals (see Fig.4A).

Despite being the first stable isotope tracer to be used by the pioneers of the technique Schonheimer and Rittenberg in the 1930s (Schoenheimer and Rittenberg 1935, 1938; Rittenberg and Schoenheimer 1937), D_2_O has been largely overlooked in favor of metabolite-specific stable or radio-labeled tracers until the last decade when its reintroduction has garnered great interest (Dufner and Previs 2003; Gasier et al. 2010; Robinson et al. 2011; Wilkinson et al. 2014). Indeed D_2_O is advantageous for a number of reasons including the possibility to quantify turnover of multiple substrate pools; indeed, our laboratory, as well as others have shown its novel utility for measuring the turnover of protein (Robinson et al. 2011; Wilkinson et al. 2014), lipids (Strawford et al. 2004), DNA (Busch et al. 2008) and glucose (Katanik et al. 2003) amongst others. By taking advantage of the relatively slow turnover rate of the body water pool (half-life ∼11 days (Mac-Donald et al. 2013; Wilkinson et al. 2014)) this also permits long-term measurements that are not possible with other techniques. For example, in a recent study (Wilkinson et al. 2014) we demonstrated the potential D_2_O has to detect changes in MPS over 2–8 days when using our highly sensitive analytical techniques, further to this its utility for measuring MPS over longer periods has also been highlighted (Robinson et al. 2011; Miller et al. 2013), with additional studies providing validation of this technique for MPS and plasma protein turnover measures (Belloto et al. 2007; Gasier et al. 2009). In the present study, we have taken this a step further. Herein, we show that in providing a single-dose of D_2_O, we are able to acutely quantify MPS over periods of ∼3 h— equivalent to what is possible with traditional AA-tracers (see Fig.4A). Moreover, using our sensitive techniques we show that D_2_O-derived FSR values yield rates of MPS that are extremely well aligned to those using traditional AA-tracer techniques in this and past studies (Mitchell et al. 2014; Phillips et al. 2014), which for myofibrillar FSR range between 0.02 and 0.07%·h^−1^ (Kumar et al. 2009; Smith et al. 2011). This engenders confidence we are accurately measuring quantitative readouts of MPS; indeed, previous studies using the D_2_O tracer technique from other groups have yielded absolute rates of MPS far out-with normal ranges (8–10%·day^−1^; equivalent to ∼0.3–0.4%·h^−1^; (Gasier et al. 2012; Lambert et al. 2015)), when measuring using far less sensitive equipment to that in the present study (GC-MS compared to GC-IRMS), therefore providing doubts as to robustness of this previous data.

Proof-of-concept that the application of these D_2_O tracers is feasible over shorter periods also requires examination of the capacity to distinguish changes in response to anabolic or for that matter, catabolic stimuli. To determine this, we elected to provide an oral bolus of EAA since these are known to be the nutritional components responsible for postprandial stimulation of tissue synthesis (in this case, MPS). We showed that the associated plasma aminoacidemia led to an expected increase in MPS using both tracers (statistically different from baseline in comparison to tracer-respective postabsorptive values), and also that there were no means-based differences between the tracers either under postabsorptive or postprandial conditions. Therefore, our results reveal that the techniques we applied are robust not only in the context of accurate quantitation of MPS but also in response to a well-defined anabolic stimuli. Collectively, these findings demonstrate the potential for D_2_O to replace AA-tracers from the perspective of: (1) studying vulnerable populations (frail elderly, adolescent, clinical populations etc.); (2) reducing study invasiveness; (3) reducing clinical inputs; and (4) reducing study costs.

Whilst there are obvious benefits to this technique, we would also like to acknowledge some potential limitations. Using means-based analysis, both the use of D_2_O and L-[ring-^13^C_6_]-phenylalanine techniques produce similar rates of MPS, with a similar increase in MPS with a 20 g EAA bolus (∼+0.02–0.03%·h^−1^; see Fig.4A). Despite the same qualitative change, on an individual basis the two techniques did not quantitatively agree in all subjects in terms of absolutes rates (see Fig.4B and C). Despite these quantitative differences, Bland–Altman plots revealed a relatively low bias between the two methods of 0.0083, which suggests that overall there were minimal differences between the methods; furthermore there was a significant correlation between the two (Fig.5B). A similar finding was also observed previously when D_2_O was compared with a phenylalanine tracer in a rat model (Gasier et al. 2009); here the authors explained that the quantitative difference may have been due to the differences in measurement periods and methodologies between the phenylalanine and D_2_O techniques (12 min and 4 h respectively), and the methodologies used for tracer administration. Furthermore, Belloto et al. (2007) compared D_2_O with radiolabeled leucine tracers for the measurement of plasma apoB100-VDL protein turnover, highlighting overall qualitatively comparable rates of turnover using the two methods similar to the present findings. In the present study, we believe that the discrepancy in the absolute rates may be due to analytical precision. Measurement precision for analysis of hydrogen using Pyrolysis-IRMS is ∼1–2 delta in our hands on the presently used equipment (Delta V Advantage, Thermo Scientific) whilst carbon measurement precision via Combustion-IRMS is ∼10 times greater than hydrogen at between 0.1 and 0.3 delta. Therefore, with such small delta shifts over 3 h for hydrogen, the measurement accuracy of this technique may affect the quantitative accuracy of the calculated rates somewhat. To combat this, one could increase the dosage of D_2_O to an appropriate level so that this measurement error is reduced, although this could introduce greater risk of adverse reaction (nausea, dizziness) in the individuals being tested, however, this could be avoided with provision of small regular doses over several hours. It should also be noted that there is no definitive methodology for performing such tracer validation studies, as each approach studied here comes with its own set of limitations and assumptions associated with the tracer and the calculations, but given this it is reassuring that there is a strong level of agreement between the two approaches.

In conclusion, here we show that D_2_O can act as an effective alternative to traditional AA-tracers when measuring MPS acutely. It only requires oral dosing of D_2_O 24 h prior to the measurement period, regular saliva samples, and appropriately positioned biopsy sampling. This reduces the need for additional invasive sampling and significantly reduces overall study costs, and subject burden, permitting measurement of MPS in clinical situations or study populations where it previously was not possible.
